# Supplementary material for: Uncovering miRNA-mRNA Regulatory Modules in Developing Xylem of Pinus massoniana via Small RNA and Degradome Sequencing
Source: Int J Mol Sci. 2021 Sep 21;22(18):10154. doi: 10.3390/ijms221810154 (PMC8472836; doi:10.3390/ijms221810154)
Supplement: Supplementary file 1 [file ijms-22-10154-s001.zip › ijms-1365557-supplementary/Additional file S2.zip!/Additional file 2/novel_122_Cluster-18410.48670_130_TPlot.pdf]

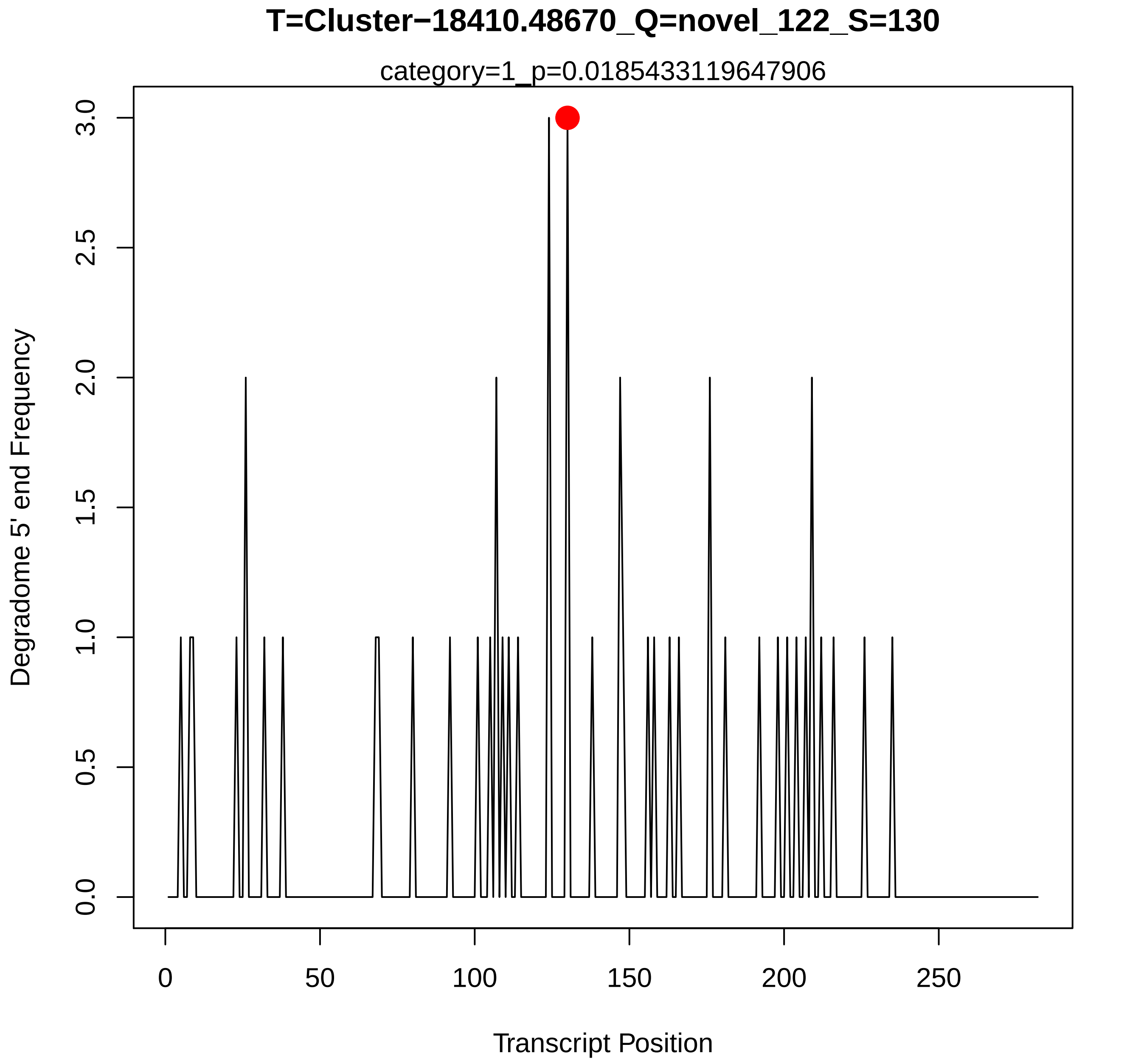
T=Cluster−18410.48670_Q=novel_122_S=130
category=1_p=0.0185433119647906
Degradome 5' end Frequency
0.0
0.5
1.0
1.5
2.0
2.5
3.0
0
50
100
150
200
250
Transcript Position
